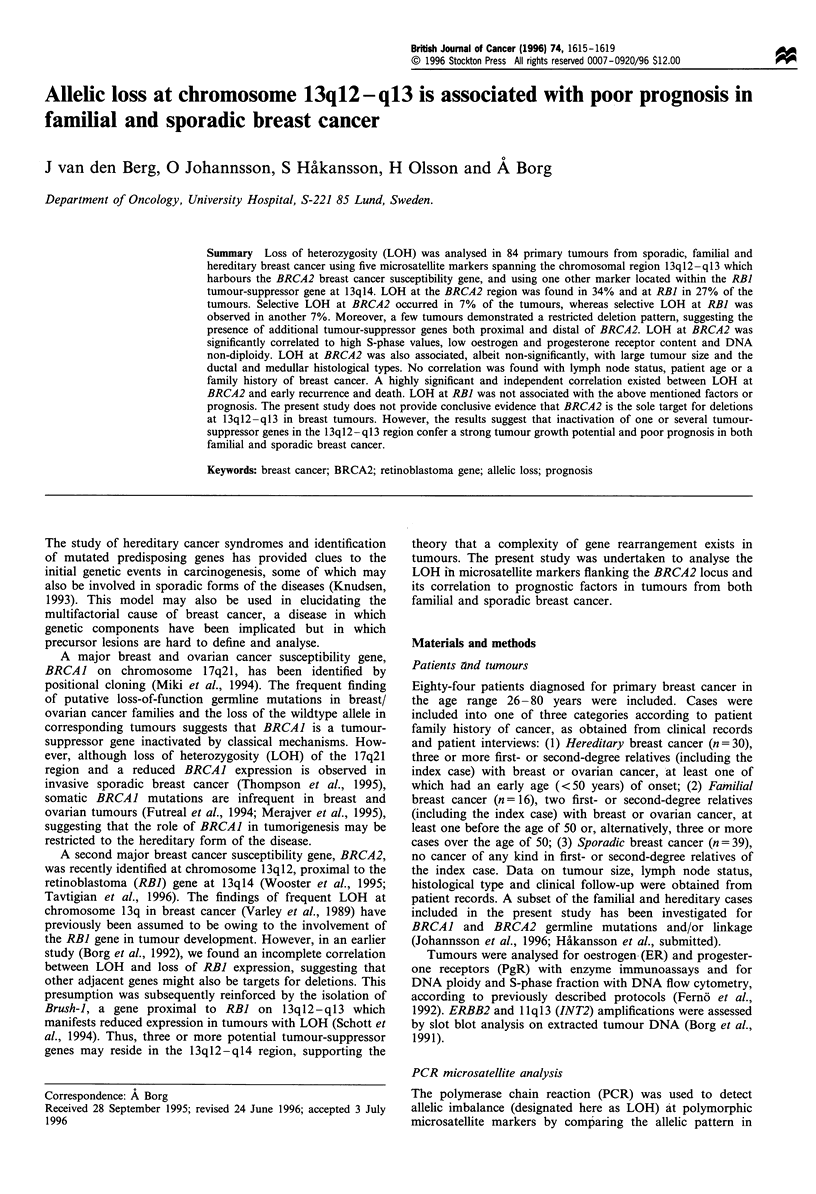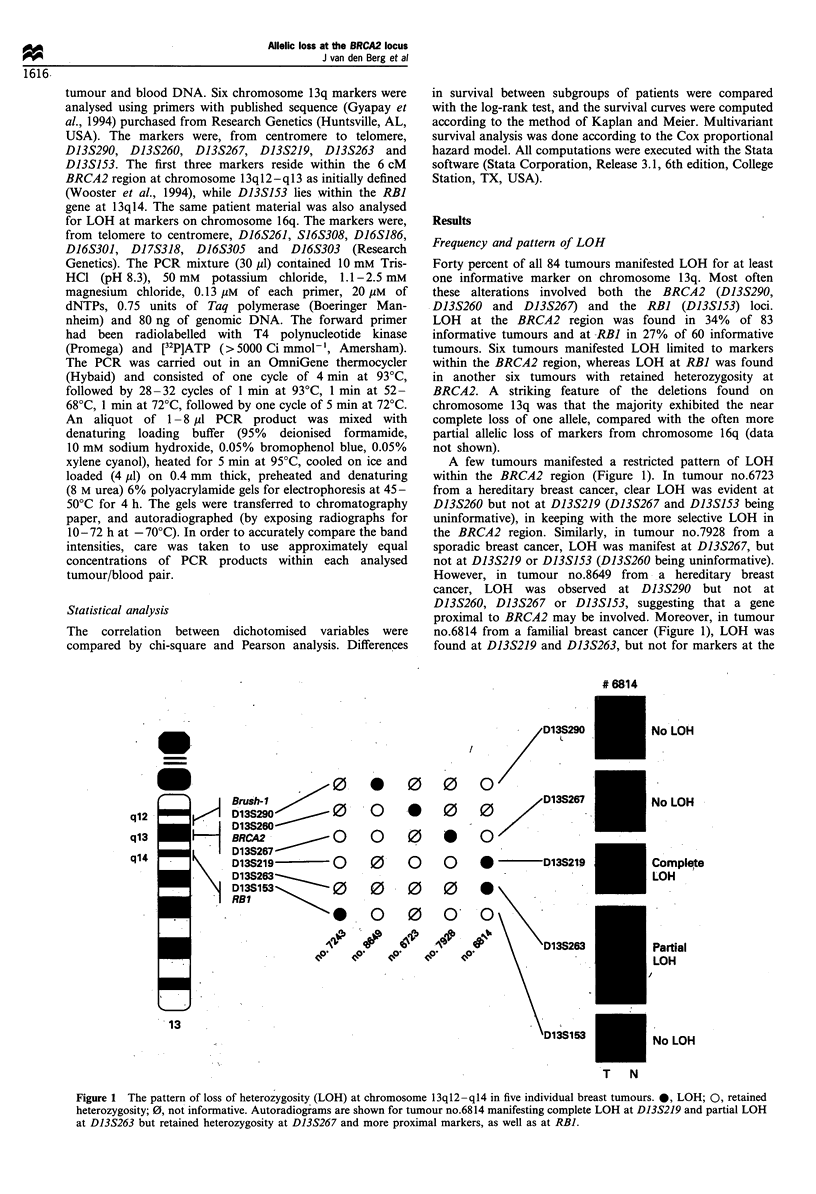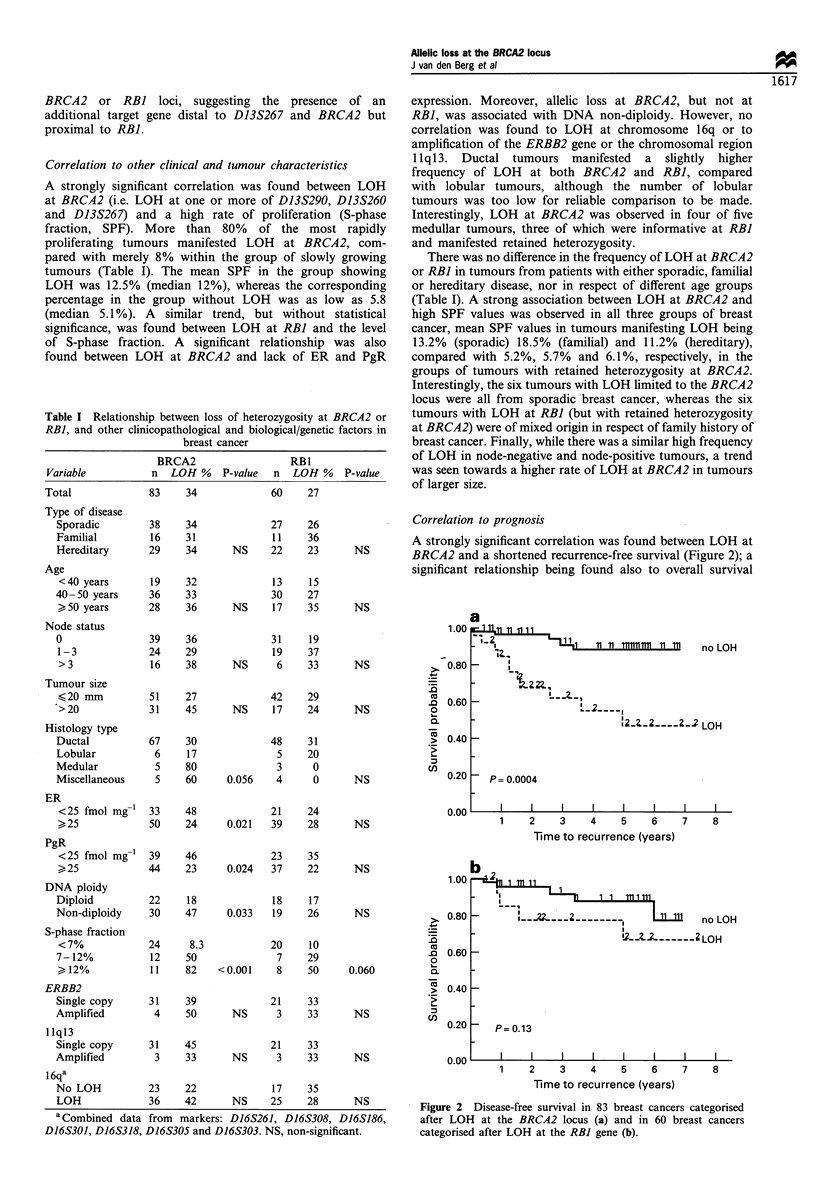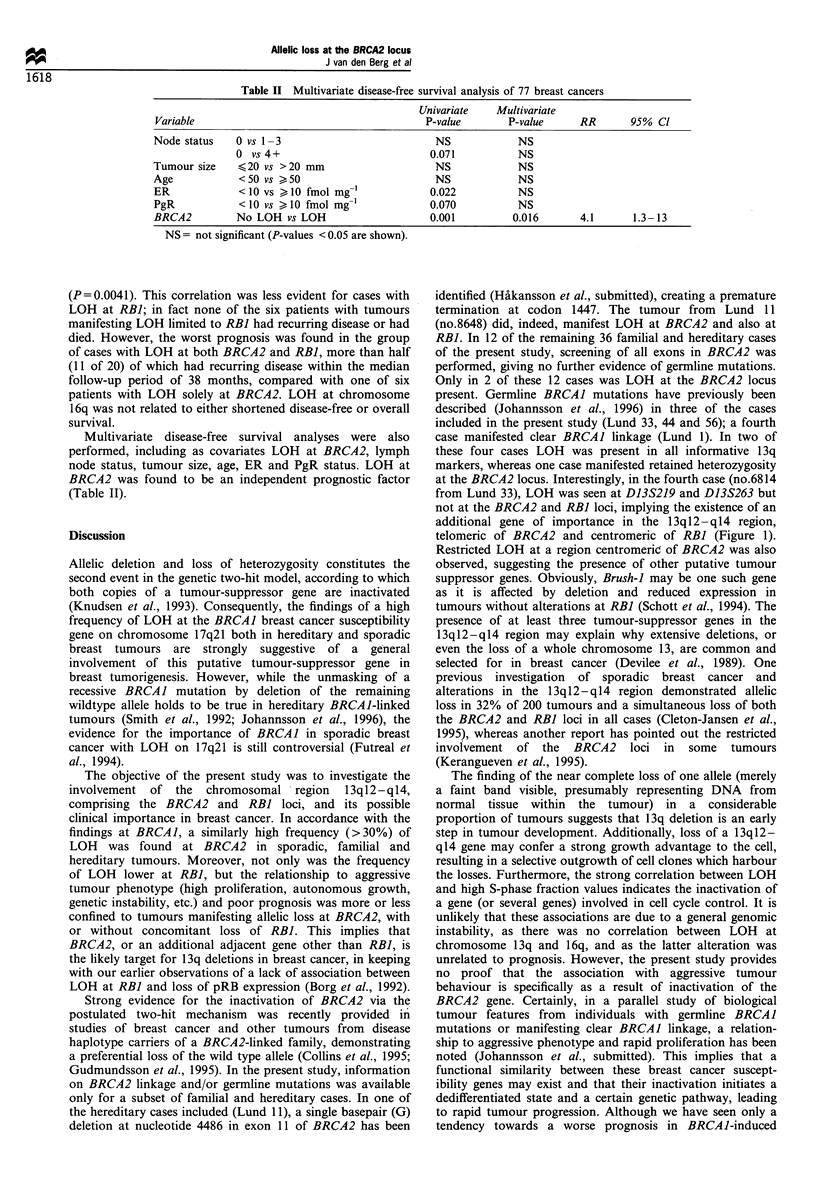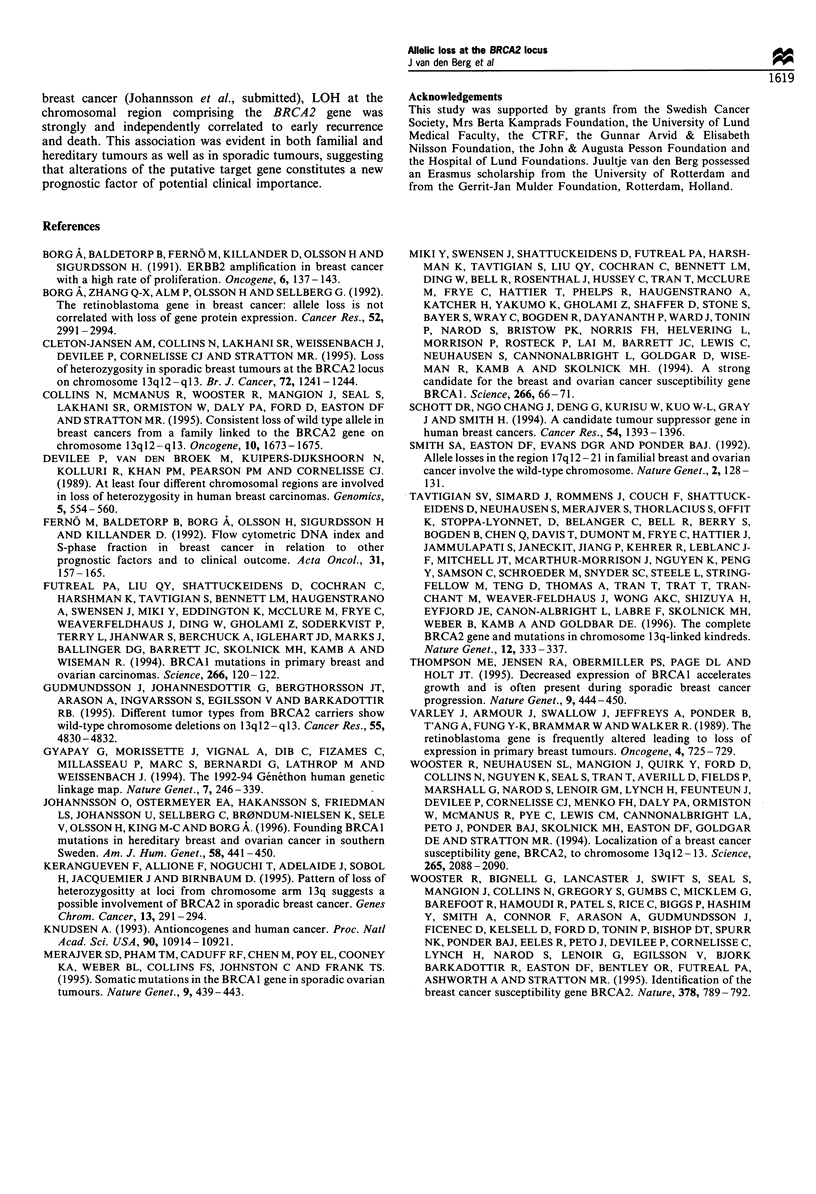# Allelic loss at chromosome 13q12-q13 is associated with poor prognosis in familial and sporadic breast cancer.

**DOI:** 10.1038/bjc.1996.597

**Published:** 1996-11

**Authors:** J. van den Berg, O. Johannsson, S. Håkansson, H. Olsson, A. Borg

**Affiliations:** Department of Oncology, University Hospital, Lund, Sweden.

## Abstract

**Images:**


British Journal of Cancer (1996) 74, 1615-1619
© 1996 Stockton Press All rights reserved 0007-0920/96 $12.00

### Allelic loss at chromosome 13q12–q13 is associated with poor prognosis in familial and sporadic breast cancer

J van den Berg, O Johannsson, S Håkansson, H Olsson and Å Borg

*Department of Oncology, University Hospital, S-221 85 Lund, Sweden.*

**Summary** Loss of heterozygosity (LOH) was analysed in 84 primary tumours from sporadic, familial and hereditary breast cancer using five microsatellite markers spanning the chromosomal region 13q12–q13 which harbours the *BRCA2* breast cancer susceptibility gene, and using one other marker located within the *RB1* tumour-suppressor gene at 13q14. LOH at the *BRCA2* region was found in 34% and at *RB1* in 27% of the tumours. Selective LOH at *BRCA2* occurred in 7% of the tumours, whereas selective LOH at *RB1* was observed in another 7%. Moreover, a few tumours demonstrated a restricted deletion pattern, suggesting the presence of additional tumour-suppressor genes both proximal and distal of *BRCA2*. LOH at *BRCA2* was significantly correlated to high S-phase values, low oestrogen and progesterone receptor content and DNA non-diploidy. LOH at *BRCA2* was also associated, albeit non-significantly, with large tumour size and the ductal and medullar histological types. No correlation was found with lymph node status, patient age or a family history of breast cancer. A highly significant and independent correlation existed between LOH at *BRCA2* and early recurrence and death. LOH at *RB1* was not associated with the above mentioned factors or prognosis. The present study does not provide conclusive evidence that *BRCA2* is the sole target for deletions at 13q12–q13 in breast tumours. However, the results suggest that inactivation of one or several tumour-suppressor genes in the 13q12–q13 region confer a strong tumour growth potential and poor prognosis in both familial and sporadic breast cancer.

**Keywords:** breast cancer; BRCA2; retinoblastoma gene; allelic loss; prognosis

The study of hereditary cancer syndromes and identification of mutated predisposing genes has provided clues to the initial genetic events in carcinogenesis, some of which may also be involved in sporadic forms of the diseases (Knudsen, 1993). This model may also be used in elucidating the multifactorial cause of breast cancer, a disease in which genetic components have been implicated but in which precursor lesions are hard to define and analyse.

A major breast and ovarian cancer susceptibility gene, *BRCA1* on chromosome 17q21, has been identified by positional cloning (Miki *et al.*, 1994). The frequent finding of putative loss-of-function germline mutations in breast/ovarian cancer families and the loss of the wildtype allele in corresponding tumours suggests that *BRCA1* is a tumour-suppressor gene inactivated by classical mechanisms. However, although loss of heterozygosity (LOH) of the 17q21 region and a reduced *BRCA1* expression is observed in invasive sporadic breast cancer (Thompson *et al.*, 1995), somatic *BRCA1* mutations are infrequent in breast and ovarian tumours (Futreal *et al.*, 1994; Merajver *et al.*, 1995), suggesting that the role of *BRCA1* in tumorigenesis may be restricted to the hereditary form of the disease.

A second major breast cancer susceptibility gene, *BRCA2*, was recently identified at chromosome 13q12, proximal to the retinoblastoma (*RB1*) gene at 13q14 (Wooster *et al.*, 1995; Tavtigian *et al.*, 1996). The findings of frequent LOH at chromosome 13q in breast cancer (Varley *et al.*, 1989) have previously been assumed to be owing to the involvement of the *RB1* gene in tumour development. However, in an earlier study (Borg *et al.*, 1992), we found an incomplete correlation between LOH and loss of *RB1* expression, suggesting that other adjacent genes might also be targets for deletions. This presumption was subsequently reinforced by the isolation of *Brush-1*, a gene proximal to *RB1* on 13q12–q13 which manifests reduced expression in tumours with LOH (Schott *et al.*, 1994). Thus, three or more potential tumour-suppressor genes may reside in the 13q12–q14 region, supporting the theory that a complexity of gene rearrangement exists in tumours. The present study was undertaken to analyse the LOH in microsatellite markers flanking the *BRCA2* locus and its correlation to prognostic factors in tumours from both familial and sporadic breast cancer.

Correspondence: Å Borg
Received 28 September 1995; revised 24 June 1996; accepted 3 July 1996

### Materials and methods

### *Patients and tumours*

Eighty-four patients diagnosed for primary breast cancer in the age range 26–80 years were included. Cases were included into one of three categories according to patient family history of cancer, as obtained from clinical records and patient interviews: (1) *Hereditary* breast cancer ($n=30$), three or more first- or second-degree relatives (including the index case) with breast or ovarian cancer, at least one of which had an early age (<50 years) of onset; (2) *Familial* breast cancer ($n=16$), two first- or second-degree relatives (including the index case) with breast or ovarian cancer, at least one before the age of 50 or, alternatively, three or more cases over the age of 50; (3) *Sporadic* breast cancer ($n=39$), no cancer of any kind in first- or second-degree relatives of the index case. Data on tumour size, lymph node status, histological type and clinical follow-up were obtained from patient records. A subset of the familial and hereditary cases included in the present study has been investigated for *BRCA1* and *BRCA2* germline mutations and/or linkage (Johannsson *et al.*, 1996; Håkansson *et al.*, submitted).

Tumours were analysed for oestrogen (ER) and progesterone receptors (PgR) with enzyme immunoassays and for DNA ploidy and S-phase fraction with DNA flow cytometry, according to previously described protocols (Fernö *et al.*, 1992). *ERBB2* and 11q13 (*INT2*) amplifications were assessed by slot blot analysis on extracted tumour DNA (Borg *et al.*, 1991).

### *PCR microsatellite analysis*

The polymerase chain reaction (PCR) was used to detect allelic imbalance (designated here as LOH) at polymorphic microsatellite markers by comparing the allelic pattern in tumour and blood DNA. Six chromosome 13q markers were analysed using primers with published sequence (Gyapay *et al.*, 1994) purchased from Research Genetics (Huntsville, AL, USA). The markers were, from centromere to telomere, *D13S290*, *D13S260*, *D13S267*, *D13S219*, *D13S263* and *D13S153*. The first three markers reside within the 6 cM *BRCA2* region at chromosome 13q12–q13 as initially defined (Wooster *et al.*, 1994), while *D13S153* lies within the *RB1* gene at 13q14. The same patient material was also analysed for LOH at markers on chromosome 16q. The markers were, from telomere to centromere, *D16S261*, *S16S308*, *D16S186*, *D16S301*, *D17S318*, *D16S305* and *D16S303* (Research Genetics). The PCR mixture (30 $\mu$l) contained 10 mM Tris-HCl (pH 8.3), 50 mM potassium chloride, 1.1–2.5 mM magnesium chloride, 0.13 $\mu$M of each primer, 20 $\mu$M of dNTPs, 0.75 units of *Taq* polymerase (Boeringer Mannheim) and 80 ng of genomic DNA. The forward primer had been radiolabelled with T4 polynucleotide kinase (Promega) and [$^{32}$P]ATP (>5000 Ci mmol$^{-1}$, Amersham). The PCR was carried out in an OmniGene thermocycler (Hybaid) and consisted of one cycle of 4 min at 93°C, followed by 28–32 cycles of 1 min at 93°C, 1 min at 52–68°C, 1 min at 72°C, followed by one cycle of 5 min at 72°C. An aliquot of 1–8 $\mu$l PCR product was mixed with denaturing loading buffer (95% deionised formamide, 10 mM sodium hydroxide, 0.05% bromophenol blue, 0.05% xylene cyanol), heated for 5 min at 95°C, cooled on ice and loaded (4 $\mu$l) on 0.4 mm thick, preheated and denaturing (8 M urea) 6% polyacrylamide gels for electrophoresis at 45–50°C for 4 h. The gels were transferred to chromatography paper, and autoradiographed (by exposing radiographs for 10–72 h at −70°C). In order to accurately compare the band intensities, care was taken to use approximately equal concentrations of PCR products within each analysed tumour/blood pair.

*Statistical analysis*

The correlation between dichotomised variables were compared by chi-square and Pearson analysis. Differences in survival between subgroups of patients were compared with the log-rank test, and the survival curves were computed according to the method of Kaplan and Meier. Multivariant survival analysis was done according to the Cox proportional hazard model. All computations were executed with the Stata software (Stata Corporation, Release 3.1, 6th edition, College Station, TX, USA).

### Results

*Frequency and pattern of LOH*

Forty percent of all 84 tumours manifested LOH for at least one informative marker on chromosome 13q. Most often these alterations involved both the *BRCA2* (*D13S290*, *D13S260* and *D13S267*) and the *RB1* (*D13S153*) loci. LOH at the *BRCA2* region was found in 34% of 83 informative tumours and at *RB1* in 27% of 60 informative tumours. Six tumours manifested LOH limited to markers within the *BRCA2* region, whereas LOH at *RB1* was found in another six tumours with retained heterozygosity at *BRCA2*. A striking feature of the deletions found on chromosome 13q was that the majority exhibited the near complete loss of one allele, compared with the often more partial allelic loss of markers from chromosome 16q (data not shown).

A few tumours manifested a restricted pattern of LOH within the *BRCA2* region (Figure 1). In tumour no.6723 from a hereditary breast cancer, clear LOH was evident at *D13S260* but not at *D13S219* (*D13S267* and *D13S153* being uninformative), in keeping with the more selective LOH in the *BRCA2* region. Similarly, in tumour no.7928 from a sporadic breast cancer, LOH was manifest at *D13S267*, but not at *D13S219* or *D13S153* (*D13S260* being uninformative). However, in tumour no.8649 from a hereditary breast cancer, LOH was observed at *D13S290* but not at *D13S260*, *D13S267* or *D13S153*, suggesting that a gene proximal to *BRCA2* may be involved. Moreover, in tumour no.6814 from a familial breast cancer (Figure 1), LOH was found at *D13S219* and *D13S263*, but not for markers at the

**Figure 1** The pattern of loss of heterozygosity (LOH) at chromosome 13q12–q14 in five individual breast tumours. ●, LOH; ○, retained heterozygosity; Ø, not informative. Autoradiograms are shown for tumour no.6814 manifesting complete LOH at *D13S219* and partial LOH at *D13S263* but retained heterozygosity at *D13S267* and more proximal markers, as well as at *RB1*.

*BRCA2* or *RB1* loci, suggesting the presence of an additional target gene distal to *D13S267* and *BRCA2* but proximal to *RB1*.

*Correlation to other clinical and tumour characteristics*

A strongly significant correlation was found between LOH at *BRCA2* (i.e. LOH at one or more of *D13S290*, *D13S260* and *D13S267*) and a high rate of proliferation (S-phase fraction, SPF). More than 80% of the most rapidly proliferating tumours manifested LOH at *BRCA2*, compared with merely 8% within the group of slowly growing tumours (Table I). The mean SPF in the group showing LOH was 12.5% (median 12%), whereas the corresponding percentage in the group without LOH was as low as 5.8 (median 5.1%). A similar trend, but without statistical significance, was found between LOH at *RB1* and the level of S-phase fraction. A significant relationship was also found between LOH at *BRCA2* and lack of ER and PgR

**Table I** Relationship between loss of heterozygosity at *BRCA2* or *RB1*, and other clinicopathological and biological/genetic factors in breast cancer

| | BRCA2 | | | RB1 | | |
|---|---|---|---|---|---|---|
| *Variable* | n | *LOH %* | P-*value* | n | *LOH %* | P-*value* |
| Total | 83 | 34 | | 60 | 27 | |
| Type of disease | | | | | | |
| Sporadic | 38 | 34 | | 27 | 26 | |
| Familial | 16 | 31 | | 11 | 36 | |
| Hereditary | 29 | 34 | NS | 22 | 23 | NS |
| Age | | | | | | |
| <40 years | 19 | 32 | | 13 | 15 | |
| 40–50 years | 36 | 33 | | 30 | 27 | |
| ⩾50 years | 28 | 36 | NS | 17 | 35 | NS |
| Node status | | | | | | |
| 0 | 39 | 36 | | 31 | 19 | |
| 1–3 | 24 | 29 | | 19 | 37 | |
| >3 | 16 | 38 | NS | 6 | 33 | NS |
| Tumour size | | | | | | |
| ⩽20 mm | 51 | 27 | | 42 | 29 | |
| >20 | 31 | 45 | NS | 17 | 24 | NS |
| Histology type | | | | | | |
| Ductal | 67 | 30 | | 48 | 31 | |
| Lobular | 6 | 17 | | 5 | 20 | |
| Medular | 5 | 80 | | 3 | 0 | |
| Miscellaneous | 5 | 60 | 0.056 | 4 | 0 | NS |
| ER | | | | | | |
| <25 fmol $mg^{-1}$ | 33 | 48 | | 21 | 24 | |
| ⩾25 | 50 | 24 | 0.021 | 39 | 28 | NS |
| PgR | | | | | | |
| <25 fmol $mg^{-1}$ | 39 | 46 | | 23 | 35 | |
| ⩾25 | 44 | 23 | 0.024 | 37 | 22 | NS |
| DNA ploidy | | | | | | |
| Diploid | 22 | 18 | | 18 | 17 | |
| Non-diploidy | 30 | 47 | 0.033 | 19 | 26 | NS |
| S-phase fraction | | | | | | |
| <7% | 24 | 8.3 | | 20 | 10 | |
| 7–12% | 12 | 50 | | 7 | 29 | |
| ⩾12% | 11 | 82 | <0.001 | 8 | 50 | 0.060 |
| *ERBB2* | | | | | | |
| Single copy | 31 | 39 | | 21 | 33 | |
| Amplified | 4 | 50 | NS | 3 | 33 | NS |
| 11q13 | | | | | | |
| Single copy | 31 | 45 | | 21 | 33 | |
| Amplified | 3 | 33 | NS | 3 | 33 | NS |
| 16q[a] | | | | | | |
| No LOH | 23 | 22 | | 17 | 35 | |
| LOH | 36 | 42 | NS | 25 | 28 | NS |

[a]Combined data from markers: *D16S261*, *D16S308*, *D16S186*, *D16S301*, *D16S318*, *D16S305* and *D16S303*. NS, non-significant.

expression. Moreover, allelic loss at *BRCA2*, but not at *RB1*, was associated with DNA non-diploidy. However, no correlation was found to LOH at chromosome 16q or to amplification of the *ERBB2* gene or the chromosomal region 11q13. Ductal tumours manifested a slightly higher frequency of LOH at both *BRCA2* and *RB1*, compared with lobular tumours, although the number of lobular tumours was too low for reliable comparison to be made. Interestingly, LOH at *BRCA2* was observed in four of five medullar tumours, three of which were informative at *RB1* and manifested retained heterozygosity.

There was no difference in the frequency of LOH at *BRCA2* or *RB1* in tumours from patients with either sporadic, familial or hereditary disease, nor in respect of different age groups (Table I). A strong association between LOH at *BRCA2* and high SPF values was observed in all three groups of breast cancer, mean SPF values in tumours manifesting LOH being 13.2% (sporadic) 18.5% (familial) and 11.2% (hereditary), compared with 5.2%, 5.7% and 6.1%, respectively, in the groups of tumours with retained heterozygosity at *BRCA2*. Interestingly, the six tumours with LOH limited to the *BRCA2* locus were all from sporadic breast cancer, whereas the six tumours with LOH at *RB1* (but with retained heterozygosity at *BRCA2*) were of mixed origin in respect of family history of breast cancer. Finally, while there was a similar high frequency of LOH in node-negative and node-positive tumours, a trend was seen towards a higher rate of LOH at *BRCA2* in tumours of larger size.

*Correlation to prognosis*

A strongly significant correlation was found between LOH at *BRCA2* and a shortened recurrence-free survival (Figure 2); a significant relationship being found also to overall survival

**Figure 2** Disease-free survival in 83 breast cancers categorised after LOH at the *BRCA2* locus (**a**) and in 60 breast cancers categorised after LOH at the *RB1* gene (**b**).

**Table II** Multivariate disease-free survival analysis of 77 breast cancers

| *Variable* | | *Univariate* P-*value* | *Multivariate* P-*value* | *RR* | *95% Cl* |
|---|---|---|---|---|---|
| Node status | 0 *vs* 1−3 | NS | NS | | |
| | 0 *vs* 4+ | 0.071 | NS | | |
| Tumour size | ⩽20 *vs* >20 mm | NS | NS | | |
| Age | <50 *vs* ⩾50 | NS | NS | | |
| ER | <10 vs ⩾10 fmol $mg^{-1}$ | 0.022 | NS | | |
| PgR | <10 *vs* ⩾10 fmol $mg^{-1}$ | 0.070 | NS | | |
| *BRCA2* | No LOH *vs* LOH | 0.001 | 0.016 | 4.1 | 1.3−13 |

NS= not significant (*P*-values <0.05 are shown).

($P$=0.0041). This correlation was less evident for cases with LOH at *RB1*; in fact none of the six patients with tumours manifesting LOH limited to *RB1* had recurring disease or had died. However, the worst prognosis was found in the group of cases with LOH at both *BRCA2* and *RB1*, more than half (11 of 20) of which had recurring disease within the median follow-up period of 38 months, compared with one of six patients with LOH solely at *BRCA2*. LOH at chromosome 16q was not related to either shortened disease-free or overall survival.

Multivariate disease-free survival analyses were also performed, including as covariates LOH at *BRCA2*, lymph node status, tumour size, age, ER and PgR status. LOH at *BRCA2* was found to be an independent prognostic factor (Table II).

### Discussion

Allelic deletion and loss of heterozygosity constitutes the second event in the genetic two-hit model, according to which both copies of a tumour-suppressor gene are inactivated (Knudsen *et al.*, 1993). Consequently, the findings of a high frequency of LOH at the *BRCA1* breast cancer susceptibility gene on chromosome 17q21 both in hereditary and sporadic breast tumours are strongly suggestive of a general involvement of this putative tumour-suppressor gene in breast tumorigenesis. However, while the unmasking of a recessive *BRCA1* mutation by deletion of the remaining wildtype allele holds to be true in hereditary *BRCA1*-linked tumours (Smith *et al.*, 1992; Johannsson *et al.*, 1996), the evidence for the importance of *BRCA1* in sporadic breast cancer with LOH on 17q21 is still controversial (Futreal *et al.*, 1994).

The objective of the present study was to investigate the involvement of the chromosomal region 13q12−q14, comprising the *BRCA2* and *RB1* loci, and its possible clinical importance in breast cancer. In accordance with the findings at *BRCA1*, a similarly high frequency (>30%) of LOH was found at *BRCA2* in sporadic, familial and hereditary tumours. Moreover, not only was the frequency of LOH lower at *RB1*, but the relationship to aggressive tumour phenotype (high proliferation, autonomous growth, genetic instability, etc.) and poor prognosis was more or less confined to tumours manifesting allelic loss at *BRCA2*, with or without concomitant loss of *RB1*. This implies that *BRCA2*, or an additional adjacent gene other than *RB1*, is the likely target for 13q deletions in breast cancer, in keeping with our earlier observations of a lack of association between LOH at *RB1* and loss of pRB expression (Borg *et al.*, 1992).

Strong evidence for the inactivation of *BRCA2* via the postulated two-hit mechanism was recently provided in studies of breast cancer and other tumours from disease haplotype carriers of a *BRCA2*-linked family, demonstrating a preferential loss of the wild type allele (Collins *et al.*, 1995; Gudmundsson *et al.*, 1995). In the present study, information on *BRCA2* linkage and/or germline mutations was available only for a subset of familial and hereditary cases. In one of the hereditary cases included (Lund 11), a single basepair (G) deletion at nucleotide 4486 in exon 11 of *BRCA2* has been identified (Håkansson *et al.*, submitted), creating a premature termination at codon 1447. The tumour from Lund 11 (no.8648) did, indeed, manifest LOH at *BRCA2* and also at *RB1*. In 12 of the remaining 36 familial and hereditary cases of the present study, screening of all exons in *BRCA2* was performed, giving no further evidence of germline mutations. Only in 2 of these 12 cases was LOH at the *BRCA2* locus present. Germline *BRCA1* mutations have previously been described (Johannsson *et al.*, 1996) in three of the cases included in the present study (Lund 33, 44 and 56); a fourth case manifested clear *BRCA1* linkage (Lund 1). In two of these four cases LOH was present in all informative 13q markers, whereas one case manifested retained heterozygosity at the *BRCA2* locus. Interestingly, in the fourth case (no.6814 from Lund 33), LOH was seen at *D13S219* and *D13S263* but not at the *BRCA2* and *RB1* loci, implying the existence of an additional gene of importance in the 13q12−q14 region, telomeric of *BRCA2* and centromeric of *RB1* (Figure 1). Restricted LOH at a region centromeric of *BRCA2* was also observed, suggesting the presence of other putative tumour suppressor genes. Obviously, *Brush-1* may be one such gene as it is affected by deletion and reduced expression in tumours without alterations at *RB1* (Schott *et al.*, 1994). The presence of at least three tumour-suppressor genes in the 13q12−q14 region may explain why extensive deletions, or even the loss of a whole chromosome 13, are common and selected for in breast cancer (Devilee *et al.*, 1989). One previous investigation of sporadic breast cancer and alterations in the 13q12−q14 region demonstrated allelic loss in 32% of 200 tumours and a simultaneous loss of both the *BRCA2* and *RB1* loci in all cases (Cleton-Jansen *et al.*, 1995), whereas another report has pointed out the restricted involvement of the *BRCA2* loci in some tumours (Kerangueven *et al.*, 1995).

The finding of the near complete loss of one allele (merely a faint band visible, presumably representing DNA from normal tissue within the tumour) in a considerable proportion of tumours suggests that 13q deletion is an early step in tumour development. Additionally, loss of a 13q12−q14 gene may confer a strong growth advantage to the cell, resulting in a selective outgrowth of cell clones which harbour the losses. Furthermore, the strong correlation between LOH and high S-phase fraction values indicates the inactivation of a gene (or several genes) involved in cell cycle control. It is unlikely that these associations are due to a general genomic instability, as there was no correlation between LOH at chromosome 13q and 16q, and as the latter alteration was unrelated to prognosis. However, the present study provides no proof that the association with aggressive tumour behaviour is specifically as a result of inactivation of the *BRCA2* gene. Certainly, in a parallel study of biological tumour features from individuals with germline *BRCA1* mutations or manifesting clear *BRCA1* linkage, a relationship to aggressive phenotype and rapid proliferation has been noted (Johannsson *et al.*, submitted). This implies that a functional similarity between these breast cancer susceptibility genes may exist and that their inactivation initiates a dedifferentiated state and a certain genetic pathway, leading to rapid tumour progression. Although we have seen only a tendency towards a worse prognosis in *BRCA1*-induced breast cancer (Johannsson *et al.*, submitted), LOH at the chromosomal region comprising the *BRCA2* gene was strongly and independently correlated to early recurrence and death. This association was evident in both familial and hereditary tumours as well as in sporadic tumours, suggesting that alterations of the putative target gene constitutes a new prognostic factor of potential clinical importance.

**Acknowledgements**

This study was supported by grants from the Swedish Cancer Society, Mrs Berta Kamprads Foundation, the University of Lund Medical Faculty, the CTRF, the Gunnar Arvid & Elisabeth Nilsson Foundation, the John & Augusta Pesson Foundation and the Hospital of Lund Foundations. Juultje van den Berg possessed an Erasmus scholarship from the University of Rotterdam and from the Gerrit-Jan Mulder Foundation, Rotterdam, Holland.

### References

BORG Å, BALDETORP B, FERNÖ M, KILLANDER D, OLSSON H AND SIGURDSSON H. (1991). ERBB2 amplification in breast cancer with a high rate of proliferation. *Oncogene*, **6**, 137–143.

BORG Å, ZHANG Q-X, ALM P, OLSSON H AND SELLBERG G. (1992). The retinoblastoma gene in breast cancer: allele loss is not correlated with loss of gene protein expression. *Cancer Res.*, **52**, 2991–2994.

CLETON-JANSEN AM, COLLINS N, LAKHANI SR, WEISSENBACH J, DEVILEE P, CORNELISSE CJ AND STRATTON MR. (1995). Loss of heterozygosity in sporadic breast tumours at the BRCA2 locus on chromosome 13q12–q13. *Br. J. Cancer*, **72**, 1241–1244.

COLLINS N, MCMANUS R, WOOSTER R, MANGION J, SEAL S, LAKHANI SR, ORMISTON W, DALY PA, FORD D, EASTON DF AND STRATTON MR. (1995). Consistent loss of wild type allele in breast cancers from a family linked to the BRCA2 gene on chromosome 13q12–q13. *Oncogene*, **10**, 1673–1675.

DEVILEE P, VAN DEN BROEK M, KUIPERS-DIJKSHOORN N, KOLLURI R, KHAN PM, PEARSON PM AND CORNELISSE CJ. (1989). At least four different chromosomal regions are involved in loss of heterozygosity in human breast carcinomas. *Genomics*, **5**, 554–560.

FERNÖ M, BALDETORP B, BORG Å, OLSSON H, SIGURDSSON H AND KILLANDER D. (1992). Flow cytometric DNA index and S-phase fraction in breast cancer in relation to other prognostic factors and to clinical outcome. *Acta Oncol.*, **31**, 157–165.

FUTREAL PA, LIU QY, SHATTUCKEIDENS D, COCHRAN C, HARSHMAN K, TAVTIGIAN S, BENNETT LM, HAUGENSTRANO A, SWENSEN J, MIKI Y, EDDINGTON K, MCCLURE M, FRYE C, WEAVERFELDHAUS J, DING W, GHOLAMI Z, SODERKVIST P, TERRY L, JHANWAR S, BERCHUCK A, IGLEHART JD, MARKS J, BALLINGER DG, BARRETT JC, SKOLNICK MH, KAMB A AND WISEMAN R. (1994). BRCA1 mutations in primary breast and ovarian carcinomas. *Science*, **266**, 120–122.

GUDMUNDSSON J, JOHANNESDOTTIR G, BERGTHORSSON JT, ARASON A, INGVARSSON S, EGILSSON V AND BARKADOTTIR RB. (1995). Different tumor types from BRCA2 carriers show wild-type chromosome deletions on 13q12–q13. *Cancer Res.*, **55**, 4830–4832.

GYAPAY G, MORISSETTE J, VIGNAL A, DIB C, FIZAMES C, MILLASSEAU P, MARC S, BERNARDI G, LATHROP M AND WEISSENBACH J. (1994). The 1992-94 Généthon human genetic linkage map. *Nature Genet.*, **7**, 246–339.

JOHANNSSON O, OSTERMEYER EA, HAKANSSON S, FRIEDMAN LS, JOHANSSON U, SELLBERG C, BRØNDUM-NIELSEN K, SELE V, OLSSON H, KING M-C AND BORG Å. (1996). Founding BRCA1 mutations in hereditary breast and ovarian cancer in southern Sweden. *Am. J. Hum. Genet.*, **58**, 441–450.

KERANGUEVEN F, ALLIONE F, NOGUCHI T, ADELAIDE J, SOBOL H, JACQUEMIER J AND BIRNBAUM D. (1995). Pattern of loss of heterozygositty at loci from chromosome arm 13q suggests a possible involvement of BRCA2 in sporadic breast cancer. *Genes Chrom. Cancer*, **13**, 291–294.

KNUDSEN A. (1993). Antioncogenes and human cancer. *Proc. Natl Acad. Sci. USA*, **90**, 10914–10921.

MERAJVER SD, PHAM TM, CADUFF RF, CHEN M, POY EL, COONEY KA, WEBER BL, COLLINS FS, JOHNSTON C AND FRANK TS. (1995). Somatic mutations in the BRCA1 gene in sporadic ovarian tumours. *Nature Genet.*, **9**, 439–443.

MIKI Y, SWENSEN J, SHATTUCKEIDENS D, FUTREAL PA, HARSHMAN K, TAVTIGIAN S, LIU QY, COCHRAN C, BENNETT LM, DING W, BELL R, ROSENTHAL J, HUSSEY C, TRAN T, MCCLURE M, FRYE C, HATTIER T, PHELPS R, HAUGENSTRANO A, KATCHER H, YAKUMO K, GHOLAMI Z, SHAFFER D, STONE S, BAYER S, WRAY C, BOGDEN R, DAYANANTH P, WARD J, TONIN P, NAROD S, BRISTOW PK, NORRIS FH, HELVERING L, MORRISON P, ROSTECK P, LAI M, BARRETT JC, LEWIS C, NEUHAUSEN S, CANNONALBRIGHT L, GOLDGAR D, WISEMAN R, KAMB A AND SKOLNICK MH. (1994). A strong candidate for the breast and ovarian cancer susceptibility gene BRCA1. *Science*, **266**, 66–71.

SCHOTT DR, NGO CHANG J, DENG G, KURISU W, KUO W-L, GRAY J AND SMITH H. (1994). A candidate tumour suppressor gene in human breast cancers. *Cancer Res.*, **54**, 1393–1396.

SMITH SA, EASTON DF, EVANS DGR AND PONDER BAJ. (1992). Allele losses in the region 17q12–21 in familial breast and ovarian cancer involve the wild-type chromosome. *Nature Genet.*, **2**, 128–131.

TAVTIGIAN SV, SIMARD J, ROMMENS J, COUCH F, SHATTUCKEIDENS D, NEUHAUSEN S, MERAJVER S, THORLACIUS S, OFFIT K, STOPPA-LYONNET, D, BELANGER C, BELL R, BERRY S, BOGDEN B, CHEN Q, DAVIS T, DUMONT M, FRYE C, HATTIER J, JAMMULAPATI S, JANECKIT, JIANG P, KEHRER R, LEBLANC J-F, MITCHELL JT, MCARTHUR-MORRISON J, NGUYEN K, PENG Y, SAMSON C, SCHROEDER M, SNYDER SC, STEELE L, STRINGFELLOW M, TENG D, THOMAS A, TRAN T, TRAT T, TRANCHANT M, WEAVER-FELDHAUS J, WONG AKC, SHIZUYA H, EYFJORD JE, CANON-ALBRIGHT L, LABRE F, SKOLNICK MH, WEBER B, KAMB A AND GOLDBAR DE. (1996). The complete BRCA2 gene and mutations in chromosome 13q-linked kindreds. *Nature Genet.*, **12**, 333–337.

THOMPSON ME, JENSEN RA, OBERMILLER PS, PAGE DL AND HOLT JT. (1995). Decreased expression of BRCA1 accelerates growth and is often present during sporadic breast cancer progression. *Nature Genet.*, **9**, 444–450.

VARLEY J, ARMOUR J, SWALLOW J, JEFFREYS A, PONDER B, T'ANG A, FUNG Y-K, BRAMMAR W AND WALKER R. (1989). The retinoblastoma gene is frequently altered leading to loss of expression in primary breast tumours. *Oncogene*, **4**, 725–729.

WOOSTER R, NEUHAUSEN SL, MANGION J, QUIRK Y, FORD D, COLLINS N, NGUYEN K, SEAL S, TRAN T, AVERILL D, FIELDS P, MARSHALL G, NAROD S, LENOIR GM, LYNCH H, FEUNTEUN J, DEVILEE P, CORNELISSE CJ, MENKO FH, DALY PA, ORMISTON W, MCMANUS R, PYE C, LEWIS CM, CANNONALBRIGHT LA, PETO J, PONDER BAJ, SKOLNICK MH, EASTON DF, GOLDGAR DE AND STRATTON MR. (1994). Localization of a breast cancer susceptibility gene, BRCA2, to chromosome 13q12–13. *Science*, **265**, 2088–2090.

WOOSTER R, BIGNELL G, LANCASTER J, SWIFT S, SEAL S, MANGION J, COLLINS N, GREGORY S, GUMBS C, MICKLEM G, BAREFOOT R, HAMOUDI R, PATEL S, RICE C, BIGGS P, HASHIM Y, SMITH A, CONNOR F, ARASON A, GUDMUNDSSON J, FICENEC D, KELSELL D, FORD D, TONIN P, BISHOP DT, SPURR NK, PONDER BAJ, EELES R, PETO J, DEVILEE P, CORNELISSE C, LYNCH H, NAROD S, LENOIR G, EGILSSON V, BJORK BARKADOTTIR R, EASTON DF, BENTLEY OR, FUTREAL PA, ASHWORTH A AND STRATTON MR. (1995). Identification of the breast cancer susceptibility gene BRCA2. *Nature*, **378**, 789–792.